# Editor's Message

**DOI:** 10.29173/jchla29581

**Published:** 2021-08-01

**Authors:** Sandra McKeown

*[Français en suite]*

To our valued readership,

I hope everyone is able to find ways to enjoy these warmer months as we live through our second summer of the pandemic. It will be interesting to see what the fall holds, as more of the population becomes fully vaccinated. Working as an academic librarian at Queen’s University in Kingston during the Covid-19 era, September marks a planned return to campus after a year and a half of working remotely.

Your JCHLA editorial team recently took time to discuss ways to streamline and improve the journal’s workflow process for current and future editors. We met three times in June to review and update editor position guides and workflow documentation. The Journal will also be trialing extended terms for the Copyeditor and Production Editor positions, to allow for a 4-month (one journal issue) overlap period to help onboard new volunteers. A big thanks to our current Copyeditor and outgoing Production Editor, Amanda Caputo and Rachel Couban, for agreeing to extend their lengths of terms for this purpose.

The August issue marks the third and final issue that the Editor-in-Chief oversees, and the near end of their 3-year term on the editorial team. Participating on the JCHLA editorial team has been one of most rewarding learning experiences I’ve had in my professional career to date. I am grateful for all the wonderful colleagues that I’ve had the chance to meet and work with during this time, and I am proud of the work we have been able to accomplish as journal editors, working group members (JCHLA Data Sharing Policy), and CHLA Board members.

In September, the Journal’s leadership transitions to the very capable hands of Alanna Campbell (Northern Ontario School of Medicine) as the new Editor-in-Chief, and Colleen Pawliuk (BC Children's Hospital Research Institute) as the new Senior Editor. With the pandemic impacting the ability of so many to take on volunteer positions like this, we are very grateful to Megan Kennedy (University of Alberta) for stepping into a 3-year term beginning as the new Junior Editor, and to Courtney Ellsworth (Saskatchewan Health Authority) for joining the team as our new Production Editor.

The August 2021 issue of the Journal includes two articles about library support for knowledge synthesis research, including the program description: “Flipping it online: re-imagining teaching searching for knowledge syntheses”, and the research article: “An environmental scan of librarian involvement in systematic reviews at Queen’s University: 2020 update”. This issue features the article: “Evolution-revolution-devolution: a short history of the provision of knowledge-based information services to Manitoba’s health professionals” and reviews the book “Meeting the Challenge of Teaching Information Literacy”. A big thanks to all of our authors and peer reviewers!

Sincerely,

**Sandra McKeown**

JCHLA/JABSC Editor-in-Chief

Email: editor@chla-absc.ca

A nos lecteurs,

J'espère que vous avez tous pu profiter des mois plus chauds en ce deuxième été de pandémie. Il sera intéressant de voir ce que l'automne nous réserve, alors qu'une plus grande partie de la population sera vaccinée. En tant que bibliothécaire universitaire à l'Université Queen's à Kingston, pendant l'ère Covid-19, le mois de septembre marquera un retour sur le campus, après un an et demi de télétravail.

Votre équipe de rédaction du Journal de l’Association des bibliothèques de la santé du Canada (JABSC) a récemment pris le temps de discuter des moyens de rationaliser et d'améliorer le processus de révision pour les actuels et futurs éditeurs. Nous nous sommes réunies trois fois en juin pour examiner et mettre à jour les lignes directrices des éditeurs ainsi que la documentation afférente . La revue va également tester des mandats prolongés pour les postes de réviseur et d’éditeur de la production, afin d’allouer une période de chevauchement de quatre mois (un numéro de la revue), facilitant ainsi l'intégration des nouveaux bénévoles. Nous remercions chaleureusement Amanda Caputo et Rachel Couban, notre réviseure actuelle et notre éditrice de la production sortante, qui ont accepté de prolonger la durée de leur mandat à cette fin.

Le numéro d'Août marque le troisième et dernier numéro supervisé par l’éditrice en chef, et la fin de son mandat de trois ans au sein de l'équipe de rédaction. Faire partie de l'équipe de rédaction du JABSC a été l'une des expériences d'apprentissage les plus enrichissantes que j'ai vécues jusqu'à présent, dans ma carrière professionnelle. Je suis reconnaissante envers tous les merveilleux collègues que j'ai eu la chance de rencontrer et avec lesquels j'ai travaillé pendant cette période. Je suis fière du travail que nous avons pu accomplir en tant qu’éditrices sr6u de la revue, membres du groupe de travail (politique de partage des données du JABSC) et membres du conseil d'administration de l'ABSC.

En Septembre, la direction de la revue passera aux mains très compétentes d'Alanna Campbell (École de médecine du Nord de l'Ontario), nouvelle éditrice en chef, et de Colleen Pawliuk (Institut de recherche de l'Hôpital pour enfants de la Colombie-Britannique), nouvelle éditrice principale. Étant donné l’impact de la pandémie sur la capacité d'un grand nombre de personnes à occuper des postes bénévoles, nous sommes très reconnaissantes à Megan Kennedy (Université de l'Alberta) d'avoir accepté un mandat de trois ans en tant que nouvelle éditrice débutante, et à Courtney Ellsworth (Saskatchewan Health Authority) de s'être jointe à l'équipe en tant que nouvelle éditrice de production.

Le numéro d'Août 2021 comprend deux articles sur le soutien des bibliothèques pour la recherche sur la synthèse des connaissances, incluant la description du programme : “Flipping it online : re-imagining teaching searching for knowledge syntheses”, et l'article de recherche : “An environmental scan of librarian involvement in systematic reviews at Queen's University : 2020 update”. Ce numéro présente l'article : : “Evolution-revolution-devolution: a short history of the provision of knowledge-based information services to Manitoba’s health professionals” ainsi qu’une critique du livre “Meeting the Challenge of Teaching Information Literacy”. Un grand merci à tous nos auteurs et pairs réviseurs !

Sincèrement,

**Sandra McKeown**

JCHLA/JABSC Rédactrice en chef

Courriel: editor@chla-absc.ca

